# Lean non‐alcoholic fatty liver disease and associated metabolic disturbance: A Saudi Arabian cross‐sectional study

**DOI:** 10.14814/phy2.14949

**Published:** 2021-07-18

**Authors:** Yasir Mohammed Khayyat

**Affiliations:** ^1^ Department of Medicine Faculty of Medicine Umm Al Qura University Makkah Saudi Arabia; ^2^ Department of Medicine International Medical Centre Jeddah Saudi Arabia

**Keywords:** diabetes mellitus, type 2, hyperlipidemias, metabolic syndrome, non‐alcoholic fatty liver disease, obesity

## Abstract

Non‐alcoholic liver disease (NAFLD) is a metabolic liver disease associated with visceral adiposity and insulin resistance. Recently, NAFLD has been described in lean individuals who additionally have impaired metabolic parameters similar to their non‐lean counterparts. We aimed to explore this further in Saudi Arabia. From 2016 to 2019, we prospectively studied a group of newly diagnosed NAFLD patients at a tertiary hospital in Saudi Arabia. Patients were classified into three groups: lean (body mass index [BMI] <25), overweight (BMI ≥25 and <30), and obese (BMI ≥30). We made comparisons between these groups on basic clinical, demographic, and laboratory parameters. In total, 1753 patients were recruited and 1262 patients met the inclusion criteria. Altogether, 159 (12.6%), 365 (29%), and 737 (58.4%) patients were in the lean, overweight, and obese categories, respectively. Lean NAFLD patients were, on average, younger than those in the overweight group (mean 49.95 ± 15.3) and had a significantly higher high‐density lipoprotein value (HDL) (mean 52.56 ± 16.27). Sex, hyperlipidemia, type 2 diabetes, and hypertension were significantly associated with BMI. Lean NAFLD patients displayed the features of metabolic syndrome including elevated glycosylated hemoglobin and abnormal lipid profile but had higher serum HDL. This is in contrast to the widely held belief that lean individuals have no dysmetabolic changes compared to overweight individuals. Recognition of this problem is essential so that lean NAFLD patients can be screened for metabolic changes and managed appropriately to prevent complications.

## Graphical abstract

Been lean individuals would not insure that a healthy metabolic profile. The study confirmed that lean individuals especially in the context of fatty liver disease have a metabolic profile close to the obese general population. Lean NAFLD has a metabolic profile with dysmetabolic findings of impaired glycosylated hemoglobin and elevated lipid profile panel.

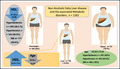

## 1 INTRODUCTION

Non‐alcoholic fatty liver disease (NAFLD) has evolved from a primary liver disorder to being proposed as a consequence of altered metabolic balance leading to a chronic, unpredictable course of hepatocyte injury, cell death, liver fibrosis, and, ultimately, cirrhosis. Biochemically, fatty liver is detected by abnormal liver function test results. Abdominal imaging of fatty liver is characterized by increased hepatic and renal echogenicity, and blurring of liver vasculature (Khov et al., 2014).

NAFLD is currently the leading cause of liver‐related morbidity and mortality and a leading cause of referral for liver transplantation. By the year 2030, the number of newly diagnosed cases of NAFLD is expected to reach 12,534,000 in Saudi Arabia and 372,000 in the United Arab Emirates; this will translate into greater liver‐related morbidity and mortality (Alswat et al., 2018).

Being overweight (body mass index [BMI] ≥25) or obese (BMI ≥30) is a risk factor for NAFLD. There is controversy surrounding the classification of obesity, especially in Asian populations, regarding whether to classify lean as BMI less than 23 or less than 25; thus far, for our local classification of obesity, the current definition follows the World Health Organization classification of obesity (2004). In Saudi Arabia, obesity is reaching epidemic proportions and the government is implementing initiatives to combat this threat. By the year 2022, the prevalence of male and female obesity is estimated to be between 12%–41% and 21%–78%, respectively (Al‐Quwaidhi et al., 2014). Currently, the prevalence of obesity in Saudi Arabia is in the range of 27.6%–55%, with men (24%–45.6%) being more obese than women (33%–38.7%) (Al‐Ghamdi et al., 2018; Al‐Hazzaa et al., 2014; Al‐Qahtani, 2019; Azzeh et al., 2017; Memish et al., 2014). Factors reported to be associated with obesity are attributed to the decreased level of activity, marital status, peer obesity, limited education, increased age, and underprivileged economic status (Albawardi et al., 2016, 2017; Khalaf et al., 2015). Understanding these complex factors with potential identifiable targets is essential for the management of patients with obesity.

However, there is an increasing occurrence of NAFLD in patients with a BMI <25 (Vos et al., 2011; Younossi et al., 2012). This “lean NAFLD” group is thought to have similar risks of liver fibrosis compared to obese NAFLD patients, but with less pronounced metabolic syndrome features (Ampuero et al., 2018; Sookoian & Pirola, 2017). This study aimed to identify the clinical and metabolic characteristics of the lean NAFLD group compared with the overweight and obese NAFLD groups at a tertiary center in the western region of Saudi Arabia.

## 2 MATERIALS AND METHODS

A prospective cross‐sectional study was conducted at our tertiary private medical center in Saudi Arabia, from 2016 to 2019, for patients who were referred from a gastroenterology clinic for the evaluation of abnormal liver function tests. These patients were subsequently diagnosed with NAFLD using ultrasound imaging. Inclusion criteria were age >15 years, abnormal liver function test results, and characteristic features of fatty liver on abdominal ultrasound imaging. Exclusion criteria were incomplete patient data; having undergone recent bariatric, endoscopic, or surgical procedures for weight loss, or other procedures that may result in intentional weight loss; age ≤15 years; other causes of chronic liver disease such as hepatitis A, B, or C, hemochromatosis, Wilson's disease, autoimmune hepatitis, primary biliary cirrhosis, primary sclerosing cholangitis; hepatic vascular diseases; active alcohol use or abuse; active malignancy; and chronic medical illnesses that would impact liver function. Patients underwent a thorough history‐taking; physical examination, with particular attention paid to the intake of herbal remedies, over‐the‐counter medications, and alcohol intake; and blood tests. Blood tests included liver function tests (after 4–6 h of fasting), glycosylated hemoglobin, and a fasting lipid profile. Adherence to diabetes, hypertension, and lipid‐lowering medications was verified through patient and family interviews and from hospital records.

The study was conducted in accordance with the principles of the Declaration of Helsinki, and all procedures were approved by the Ethics committee of the International Medical Centre (approval no.2019‐11‐115). Written informed consent was obtained from all participants prior to the start of the study.

### 2.1 Statistical analysis

For descriptive analyses, mean and standard deviations (SD) were used. Comparisons were made between the three BMI categories: lean (BMI <25), overweight (BMI ≥25 and <30), and obese (BMI ≥30). The one‐way analysis of variance (ANOVA) was used for normally distributed data and the Kruskal–Wallis test was used for non‐parametric data. Furthermore, a post hoc pairwise comparison with Bonferroni correction was performed for variables with a significant result in the one‐way ANOVA. Relationships between the BMI categories and categorical variables were analyzed using the chi‐square test of independence. A *p* value of < .05 was considered statistically significant. Statistical analyses were performed using IBM SPSS Statistics for Windows, Version 26.0 (IBM Corp).

## 3 RESULTS

A total of 1753 patients were recruited during the study period, with 1262 (72.0%) patients included in the final analyses. We excluded 491 patients for the following reasons: 103 had incomplete data, 185 had chronic hepatitis B, 71 had chronic hepatitis C, 66 underwent weight management surgery, 11 had active neoplastic disorders, 33 had coexisting medical conditions that cause liver function test alterations, 8 patients were using hepatotoxic medications, and finally 13 subjects died during the study period. The study cohort was further divided into three groups according to BMI categories: lean (159, 12.6%), overweight (365, 29%), and obese (737, 58.4%).

Descriptive analyses of anthropometric features, liver function tests, lipid profiles, and glycosylated hemoglobin tests are presented in Tables 1 and 2. Of note, the younger age group and those with lower glycosylated hemoglobin level were further analyzed. Associations between BMI and metabolic parameters including liver function tests, lipid profile, and glycosylated hemoglobin are shown in Table 3. Age was the only variable that was not normally distributed (Table 4). For high‐density lipoprotein (HDL), a significant difference was observed between lean and overweight patients (*p* = .018), that is, lean patients (Mean = 52.56, SD = 16.27) had a significantly higher HDL than overweight patients (Mean = 47.30, SD = 16.96). The effect size for HDL (using eta squared) was small (η^2^ = 0.01). There was a significant difference between lean and obese patients in terms of age (*p* = .042). On average, lean patients were younger (Mean = 49.95, SD = 15.34, Mean = 53.00) than obese patients (Mean = 53.34, SD = 13.43, Mean = 55.00). The effect size (using epsilon squared) was small (ε^2^ = 0.01). However, upon studying the correlation between age and serum HDL, there was no significant association (*p* = −.03, by Spearman correlation) observed within the entire study group or within any of the BMI categories.

**TABLE 1:** Metabolic findings in the cohort according to BMI class

Variable	Lean (*n* = 159)		Overweight (*n* = 365)		Obese (*n* = 737)	
	Mean	SD	Mean	SD	Mean	SD
Age (years)	49.95	15.34	51.34	14.33	53.34	13.43
BMI	23.14	1.95	27.70	1.71	35.38	4.62
HbA1C (%)	6.07	1.41	6.51	1.61	7.16	15.48
ALT (U/L)	37.14	66.48	32.52	32.16	30.73	30.72
AST (U/L)	28.30	23.81	26.44	26.96	25.04	20.91
GGT (U/L)	60.40	81.59	56.61	81.28	60.40	118.17
ALKP (U/L)	89.56	52.69	79.77	43.69	82.73	38.86
T. Bil (mg/dl)	0.74	1.43	0.81	1.61	0.63	1.08
D. Bil (mg/dl)	0.35	0.60	0.40	1.06	0.29	0.65
T. Chol (mg/dl)	182.07	48.19	172.69	49.50	175.03	47.37
LDL (mg/dl)	118.84	42.12	114.81	42.00	115.38	41.05
TG (mg/dl)	118.69	79.73	135.74	88.66	132.65	88.56
HDL (mg/dl)	52.56	16.27	47.30	16.96	48.49	16.50

Abbreviations: ALKP, alkaline transferase; ALT, alanine aminotransferase; AST, aspartate aminotransferase; D. Bil, direct bilirubin; GGT, gamma glutamate transferase; HDL, high‐density lipoprotein; LDL, low‐density lipoprotein; SD, standard deviation; T. Bil, total bilirubin; T. Chol, total cholesterol; TG, triglyceride.

**TABLE 2:** Frequency of group characteristics and their metabolic diseases

Variable	Lean	Overweight	Obese
Sex			
Female	61 (38.4%)	142 (38.9%)	359 (48.7%)
Male	98 (61.6%)	223 (61.1%)	378 (51.3%)
Metabolic disorders			
Hyperlipidaemia	76 (47.8%)	205 (56.2%)	457 (62.0%)
Diabetes mellitus	50 (31.4%)	171 (46.8%)	405 (55.0%)
Hypertension	50 (31.4%)	144 (39.5%)	333 (45.2%)

**TABLE 3:** Comparison between BMI and metabolic parameters^a^

Variable	*F*	*df*	*p*
HbA1C	0.49	2, 881	.611
ALT	1.75	2, 1128	.174
AST	1.24	2, 1082	.290
GGT	0.14	2, 1024	.871
ALKP	2.38	2, 1022	.093
T. Bil	1.96	2, 1020	.141
D. Bil	2.03	2, 1017	.132
T. Chol	1.48	2, 946	.227
LDL	0.39	2, 947	.679
TG	1.51	2, 949	.222
HDL	3.84	2, 924	.022

Abbreviations: ALKP, alkaline transferase; ALT, alanine aminotransferase; ANOVA, analysis of variance; AST, aspartate aminotransferase; D. Bil, direct bilirubin; GGT, gamma glutamate transferase; HDL, high‐density lipoprotein; LDL, low‐density lipoprotein; T. Bil, total bilirubin; T. Chol, total cholesterol; TG, triglyceride.

^a^ One‐way ANOVA was used; with *p* value of < .05 as statistically significant.

**TABLE 4:** Comparison between BMI and age^a^

Variable	*H*	*df*	*p*
Age	8.80	2	.012

Abbreviation: BMI, body mass index.

^a^ Using Kruskal–Wallis test; with *p* value of < .05 as statistically significant.

Sex, hyperlipidemia, diabetes, and hypertension were significantly associated with the different BMI categories (Table 5). The majority of overweight and obese patients had hyperlipidemia. Among obese patients, more than half were diabetic. A majority of lean (103, 64.8%) and overweight (198, 54.2%) participants had normal blood pressure.

**TABLE 5:** Relationship between BMI class and the metabolic disorders in the cohort^a^

Variable	*χ* ^2^	*df*	*p*	Cramer's *V*
Sex	12.34	2	.002	0.10
Hyperlipidemia	14.09	2	.001	0.11
Diabetes	33.25	2	<.001	0.17
Hypertension	12.20	2	.002	0.10

Abbreviation: BMI, body mass index.

^a^ Relationship was studied using chi‐square test; with *p* value of < .05 as statistically significant.

## 4 DISCUSSION

The increasing occurrence of NAFLD in lean individuals has distorted the previous assumption of this disease being primarily associated with obesity. Recently, NAFLD has evolved from being considered primarily a liver disorder to an understanding that it is a disorder of metabolism. A new name that incorporates this change has been suggested—metabolic‐associated fatty liver disease (MAFLD) (Balmer & Dufour, 2011; Younossi et al., 2020). This change should lead to an understanding that a multidisciplinary approach is needed with regard to MAFLD management, including input from clinical nutritionists, endocrinologists, metabolic experts, and hepatologists. Being a lean individual does not necessarily confer a healthy body nor imply protection from serious chronic metabolic changes. In addition, visceral adiposity leads to a chronic inflammatory process, resulting in steatosis and, ultimately, fibrotic changes in the liver.

Of the 1262 NAFLD patients who participated in our study, 159 (12.6%) belonged to the lean category with a BMI <25 and the remaining were obese or overweight. These numbers agree with the high prevalence of obesity in the country as reported in the introduction. However, in Asia, there is an ongoing debate about adopting a lower BMI to define overweight (BMI 23–25) and obesity parameters due to a higher prevalence of central obesity in individuals with lower BMI values (Kumar et al., 2013). This trend can be explained by the alteration in lipid metabolism that is observed in patients with NAFLD.

Advanced steatosis patients with grades 2 and 3 were found to have higher concentrations of small dense LDL (sd‐LDL) that play a part in atherogenesis (Zimmermann et al., 2011). Oxidized lipoprotein lipids' (oxLDL/oxHDL) ratio is linked to the development of NAFLD in middle‐aged adults (Kaikkonen et al., 2016). The concentration of non‐HDL‐cholesterol (non‐HDL‐C), that is the difference between total cholesterol concentration and high‐density lipoprotein concentration (T.C‐HDL), is found to be a strong predictor for the development of NAFLD (Zelber‐Sagi et al., 2014). The lean NAFLD patients had a similar metabolic profile as their overweight and obese counterparts. High glycosylated hemoglobin and lipid profiles were observed in the three groups being studied, although only serum HDL level was significantly higher in lean patients. Similar observations were made in a systematic review by Sookonian et al. (Sookoian & Pirola, 2018).

Despite the lack of a significant difference, observed in our study, between age and serum HDL, several researchers have studied the degree of relation between age and serum HDL. In advanced hepatic fibrosis patients using noninvasive measurement (BARD score), it was found that older age and higher serum HDL were independent predictors for advanced liver fibrosis (Klisic et al., 2019). Additionally, Trojak et al. from Poland reported serum HDL as a negative predictor for the development of NAFLD in middle‐aged men (Trojak et al., 2013).

Furthermore, with increasing age, serum HDL is influenced by the metabolic phenotype of the individuals and their dietary habits, such as alcohol consumption among middle and older age groups. Moderate alcohol consumption leads to a slow increase in the levels of serum HDL, total cholesterol, and serum triglycerides. Endothelial lipase (EL), which is involved in HDL metabolism, progressively increases with the degree of liver steatosis and the presence of metabolic disorders (Babak & Bashkirova, 2018). Petta et al. from Italy found that increased age in the higher tertiles above 54 affects the degree of liver fibrosis, especially with low serum HDL and impaired fasting glucose regardless of visceral obesity (Petta et al., 2017). However, in our study, patients in the lean NAFLD group were younger than those in the other groups.

Impaired glycemic control in lean NAFLD groups has been observed in other studies. Fracanzi et al., from Spain, reported that in their younger lean NAFLD group, there was a high risk of diabetes mellitus, especially in men, and they were more likely to develop non‐alcoholic steatohepatitis (Fracanzani et al., 2017). In Salzburg, Austria, Feldman et al. reported that lean NAFLD patients had greater impairment of glucose control compared to that in the non‐lean NAFLD group (Feldman et al., 2017). A large follow‐up study in Asia reported a higher incidence of type 2 diabetes mellitus and lower serum HDL in non‐lean NAFLD patients; however, no significant differences were observed in the occurrence of metabolic comorbidities between lean and non‐lean groups, and the annual incidence of lean NAFLD during the 7‐year follow‐up was 4.1% (Niriella et al., 2019).

Cardiometabolic risk was reported to be higher in young and middle‐aged individuals in Saudi Arabia, as noted by the positive correlation between waist circumference of men and serum levels of glucose, total cholesterol, and low‐density lipoprotein (LDL) (Abulmeaty et al., 2017). A recent large systematic review and meta‐analysis by Ye et al. estimated an increased incidence of all‐cause mortality, liver‐related mortality, and cardiovascular mortality of 12, 4, and 4 per 1000 person‐years, respectively. The review estimated the diagnosis of new‐onset diabetes mellitus, hypertension, and cardiovascular disease of the order of 13, 56, and 19 per 1000 person‐years, respectively (Ye et al., 2020). Therefore, people with high BMI and those with a lean BMI should be screened and treated for the components of metabolic syndrome, especially if associated with the presence of NAFLD. Our study provides evidence that, in Saudi Arabia, we should develop a consensus to use the term MAFLD and thereby recognize that screening for components of the metabolic syndrome is essential for all patients diagnosed with this disease. Efforts to incorporate strategies to detect NAFLD with abdominal ultrasound in addition to early screening for impaired glucose and lipid regulation is essential. Therefore, being a lean individual does not confer a healthy body.

It is challenging to diagnose NAFLD in lean individuals in the absence of a liver biopsy (Kumar & Mohan, 2017). Liver biopsy as a confirmatory diagnostic feature was not included in our study due to the invasiveness of the procedure, its high risk of complications, and lack of acceptance by patients. Lack of liver biopsy for the diagnosis of NAFLD is a limitation of this study; hence, a comparison of biopsy reports between the groups was not possible. Additionally, it would have been useful to compare the different BMI categories for the markers of insulin resistance, such as homeostatic model assessment for insulin resistance (HOMA‐IR), and to follow the patients over time to identify those with subsequent cardiovascular and hepatic complications. Indeed, it would be valuable if we could develop surrogate markers in future studies as these would indicate the increased likelihood of these complications arising.

In conclusion, lean NAFLD is a category of liver disease with similar disease manifestation, albeit less severe, with associated metabolic profile as similar to those seen in obese and overweight NAFLD patients. Lean NAFLD patients warrant prompt recognition and early management if we are to improve their outcomes.

## CONFLICT OF INTEREST

The authors have no conflicts of interest to disclose.

## AUTHOR CONTRIBUTION

Yasir Mohammed Khayyat undertakes design, data collection, drafting, critical revision, and final approval of the manuscript. Ms. Malogorzata Jakubowska performed the statistical analysis.
